# Evaluation of the suitability of a partially defatted black soldier fly (*Hermetia illucens* L.) larvae meal as ingredient for rainbow trout (*Oncorhynchus mykiss* Walbaum) diets

**DOI:** 10.1186/s40104-017-0191-3

**Published:** 2017-07-01

**Authors:** M. Renna, A. Schiavone, F. Gai, S. Dabbou, C. Lussiana, V. Malfatto, M. Prearo, M. T. Capucchio, I. Biasato, E. Biasibetti, M. De Marco, A. Brugiapaglia, I. Zoccarato, L. Gasco

**Affiliations:** 1 Department of Agricultural, Forest and Food Sciences, University of Torino, Largo P. Braccini 2, 10095 Grugliasco, TO Italy; 2 Department of Veterinary Sciences, University of Torino, Largo P. Braccini 2, 10095 Grugliasco, TO Italy; 3 Institute of Science of Food Production, National Research Council, Largo P. Braccini 2, 10095 Grugliasco, TO Italy; 4 Veterinary Medical Research Institute for Piedmont, Liguria and Aosta Valley, Via Bologna 148, 10154 Torino, Italy

**Keywords:** Animal performance, Apparent digestibility coefficient, Carnivorous fish, Fatty acids, Fishmeal substitution, Insect meal

## Abstract

**Background:**

Two trials were performed to evaluate a partially defatted *Hermetia illucens* (HI) larvae meal as potential feed ingredient in rainbow trout (*Oncorhynchus mykiss* Walbaum) diets. In the first trial, 360 trout (178.9 ± 9.8 g of mean initial body weight) were randomly divided into three experimental groups (4 tanks/treatment, 30 fish/tank). The fish were fed for 78 days with isonitrogenous, isolipidic and isoenergetic diets containing increasing levels of HI, on as fed basis: 0% (HI0, control diet), 25% (HI25) and 50% (HI50) of fish meal substitution, corresponding to dietary inclusion levels of 0, 20% and 40%. In the second trial, 36 trout (4 tanks/treatment, 3 fish/tank) were used to evaluate the in vivo apparent digestibility coefficients (ADC) of the same diets used in the first trial.

**Results:**

Survival, growth performance, condition factor, somatic indexes, and dorsal fillet physical quality parameters were not affected by diet. The highest dietary inclusion of HI larvae meal increased dry matter and ether extract contents of trout dorsal fillet. The use of HI larvae meal induced a decrease of valuable polyunsaturated fatty acids (PUFA) even if differences were only reported at the highest level of HI inclusion. The insect meal worsened the lipids health indexes of the same muscle. Dietary inclusion of insect meal did not alter the villus height of the fish. No differences were found among treatments in relation to ADC of ether extract and gross energy, while ADC of dry matter and crude protein were higher in HI25 if compared to HI50.

**Conclusions:**

The obtained results showed that a partially defatted HI larvae meal can be used as feed ingredient in trout diets up to 40% of inclusion level without impacting survival, growth performance, condition factor, somatic indexes, dorsal fillet physical quality parameters, and intestinal morphology of the fish. However, further investigations on specific feeding strategies and diet formulations are needed to limit the observed negative effects of the insect meal on the FA composition of dorsal muscle.

## Background

In the period 2000–2012 the world food fish aquaculture production expanded at a rate of 6.2% per year [1]. This trend is expected to continue as the demand for fish products will increase, following the raise of the world population expected by 2050. Fishmeal (FM) is considered as the optimal protein source in fish feeds. However, the availability of wild harvested marine fish stocks for FM production is decreasing and conventionally used proteins have been claimed to be no longer sustainable from both economic and ecologic points of view [1]. For these reasons, research has actively worked to deliver fish feed formulations containing alternative protein sources. The most commonly used ones are plant protein sources (such as oilseed meals, cereal proteins and grain legumes) and Processed Animal Proteins (PAPs) derived from animal by-products (poultry meal, hydrolyzed poultry feathers, blood meal). As far as the former are concerned, some adverse effects on performances or intestinal integrity have been reported [2, 3] even if technological advances in plant raw material processing and the increased knowledge on fish requirements have allowed the formulation of fish feeds mainly based on plant proteins [3]. PAPs provide good quantities of essential amino acids even if methionine, lysine, histidine, isoleucine and tryptophan could be limiting depending on the drying or cooking methodologies followed during manufacturing [4].

Among PAPs, interest is recently turned to insect meals as they are considered promising and sustainable protein [5, 6] or lipid [7] sources for monogastric animals feeds. In a context of resource scarcity and population growth, insect meals could represent a precious alternative to FM in aquaculture feeds [6], also considering that freshwater carnivorous fish eat insects in their natural environment.

Interesting results have already been reported using insect meals as FM substitute in the diets of some fish species [8–13]. Investigations have also been performed as far as sensory aspects [9, 14] and consumer acceptance are concerned [15] with promising results. Nevertheless, potential hurdles have also been highlighted (toxicity of insects through bioaccumulation, deficiencies in amino acids (AA) or long chain fatty acids (FA), chitin content, palatability, digestibility) [6]. To date, the price of insect meals is not yet competitive due to low produced quantities [16]. However, as on December 2016 the EU Commission approved the use of PAPs derived from insect in aquaculture feeds, a huge development of this market is expected in next years. Insects often accumulate fat, especially during their immature stages [6]. Full-fat insect meals contain high amounts of lipids, which are difficult to be managed by the feed industry due to overmuch energy, proneness to oxidation and a decrease in pellet stability [6]. These characteristics can therefore limit the use of insect meals, as replacement of FM, in aquafeeds. Defatting insect meals can be a solution to provide high insect protein meals for animal nutrition, and insect fat for animal nutrition [17] and other purposes (e.g., biodiesel production) [18]. The defatted meal, being richer in crude protein (CP) than soybean meal (SBM), could find a place as a protein-rich source in fish diets. Till now few researchers have dealt with the use of defatted insect meals in fish nutrition [12, 19].

Among insects, black soldier fly (*Hermetia illucens* L.) is a very promising species to be used in aquaculture nutrition. Some research using fish fed different dietary inclusion levels of *H. illucens* (HI) larvae meals has reported growth performances in line with those of fish fed conventional protein sources (mainly FM or SBM). Newton et al. [20] reported similar weight gain for channel catfish (*Ictalurus punctatus* Rafinesque) fingerlings fed diets containing up to 30% of full-fat HI prepupae larvae meal. Including 15% of a full-fat HI prepupae meal, St-Hilaire et al. [8] were able to substitute 25% of FM without negative effects on rainbow trout (*Oncorhynchus mykiss* Walbaum) growth performance. In a trial using diets formulated to contain 18 and 36% of HI meal from prepupae reared on substrates enriched with fish by-products, Sealey et al. [9] obtained similar growth performance of rainbow trout fed a control diet containing anchovy meal (substitution level: 25 and 50% on dry weight basis). Increased levels of n3 polyunsaturated fatty acids (PUFA) contents were reported in the fillets of the fish fed HI reared on substrates enriched with fish by-products. In juvenile turbot (*Psetta maxima* L.), no significant effects on feed intake and feed conversion ratio (FCR) were observed with a diet containing up to 33% of full-fat HI larvae meal [19]. In Atlantic salmon (*Salmo salar* L.), two different HI larvae meals, varying in their protein and fat contents, were tested in partial or total substitution of FM; the FM replacement led to controversial results depending on the type of HI meal used [12]. In the same trial, good protein and lipid digestibility was found.

Digestive capacity and histology of liver and gastrointestinal tract are usually investigated in fish when dietary modifications occur. Until now only one study on the effects of dietary HI larvae on liver, kidney, mid- and hind-intestine histology in Atlantic salmon has been performed [12]. Intestinal morphology is considered the main indicator of gut health and functional status, and it is generally assessed through morphometric measurements of the crypts and villi [21]. The effects of dietary modifications on the intestinal morphology using morphometric investigations have been evaluated in several livestock species, such as poultry [22], pigs [23] and rabbits [24], while in salmonids only few works are currently available [25].

In EU, the recent authorization of insect PAPs in aquaculture feeds requires further investigations to fully assess the potential inclusion of these innovative raw materials as well as the implications on the productive indexes and product quality parameters. So far, no investigations on rainbow trout have been performed using defatted HI meals and based on the above reported background, this research evaluated the effects of a partially defatted HI larvae meal on in vivo digestibility, growth performance, condition factor, somatic indexes, fillet physical and chemical quality, and intestinal morphology of rainbow trout.

## Methods

The trial was conducted at the Experimental Facility of the Department of Agricultural, Forest, and Food Sciences (DISAFA) of the University of Torino (Italy). The experimental protocol was designed according to the guidelines of the current European and Italian laws on the care and use of experimental animals (European directive 86,609/EEC, put into law in Italy with D.L. 116/92).

### Diets

A partially defatted HI larvae meal obtained processing larvae reared on vegetable by-products substrate was purchased from Hermetia Deutschland GmbH & Co. KG (Baruth/Mark, Germany) and used in the trial. HI larvae meal was partially defatted with a mechanical process performed using high pressure and without solvents. No other information was provided by the producer on substrate or processing methodologies as they are considered confidential. Three experimental diets were formulated to be isonitrogenous (crude protein – CP: about 45 g/100 g dry matter –DM), isolipidic (ether extract – EE: about 15 g/100 g DM), and isoenergetic (gross energy – GE: about 22 MJ/kg DM). The diets were obtained including, as fed basis, increasing levels of HI larvae meal in substitution of 0% (HI0), 25% (HI25), and 50% (HI50) of FM, corresponding to dietary inclusion levels of 0%, 20% and 40%. Due to the different chemical composition of HI compared to FM, and in order to maintain diets isonitrogenous, isolipidic and isoenergetic, with the increase of HI inclusion in the diets some other dietary ingredients (fish oil and wheat bran) were modified.

The experimental feeds were prepared at the DISAFA Experimental Facility. The grounded ingredients and fish oil were thoroughly mixed; water was then added to the mixture to attain an appropriate consistency for pelleting. Pellets were obtained using a 2.5 mm die meat grinder and dried at 50 °C for 48 h. The diets were stored in dark bags at −20 °C until utilisation. The ingredients of the experimental diets are reported in Table 1.

**Table 1:** Ingredients and proximate composition of HI larvae meal and experimental diets

Items	HI larvae meal	HI0	HI25	HI50
Ingredients, g/kg				
FM (Chile, super prime)^a^	-	600	450	300
HI larvae meal^b^		0	200	400
Wheat meal	-	40	40	40
Wheat bran	-	90	60	30
Fish oil	-	90	70	50
Starch gelatinized, D500	-	150	150	150
Mineral mixture^c^	-	15	15	15
Vitamin mixture^d^	-	15	15	15
Proximate composition^e^				
DM, g/100 g	94.18	96.07	94.93	95.63
CP, g/100 g DM	55.34	45.20	44.86	45.00
EE, g/100 g DM	17.97	15.86	15.74	15.81
Ash, g/100 g DM	7.12	11.40	11.43	10.11
Chitin, g/100 g DM	5.00	-	1.05	2.09
NFE, g/100 g DM^f^	14.57	27.54	26.92	26.99
Gross energy, MJ/kg DM^g^	24.37	21.71	22.35	22.60

*Abbreviations*: *HI Hermetia illucens*, *FM* fish meal, *DM* dry matter, *CP* crude protein, *EE* ether extract, *NFE* Nitrogen Free Extracts

^a^Fish meal was purchased from Corpesca S.A. (Santiago, Chile). Proximate composition (% as-fed basis): 90.4 DM; 66.7 CP; 8.3 EE; 14.9 Ash

^b^ *Hermetia illucens* larvae meal purchased from Hermetia Deutschland GmbH & Co. KG (Baruth/Mark, Germany)

^c^Mineral mixture (g or mg/kg diet): bicalcium phosphate 500 g, calcium carbonate 215 g, sodium salt 40 g, potassium chloride 90 g, magnesium chloride 124 g, magnesium carbonate 124 g, iron sulfate 20 g, zinc sulfate 4 g, copper sulfate 3 g, potassium iodide 4 mg, cobalt sulfate 20 mg, manganese sulfate 3 g, sodium fluoride 1 g (Granda Zootecnici, Cuneo, Italy)

^d^Vitamin mixture (IU or mg/kg diet): DL-αtocopherolacetate, 60 IU; sodium menadione bisulfate, 5 mg; retinylacetate, 15,000 IU; DL-cholecalciferol, 3000 IU; thiamin, 15 mg; riboflavin, 30 mg; pyridoxine, 15 mg; Vitamin B_12_, 0.05 mg; nicotinic acid, 175 mg; folic acid, 500 mg; inositol, 1000 mg; biotin, 2.5 mg; calcium panthotenate, 50 mg; choline chloride, 2000 mg (Granda Zootecnici, Cuneo, Italy)

^e^Values are reported as mean of duplicate analyses

^f^Calculated as 100 − (CP + EE + Ash + Chitin)

^g^Determined by calorimetric bomb

### Chemical analyses of feeds

The proximate composition and energy level of the HI larvae meal and of the experimental diets are shown in Table 1. Feed samples were ground using a cutting mill (MLI 204; Bühler AG, Uzwil, Switzerland) and analysed for DM (AOAC #934.01), CP (AOAC #984.13) and ash (AOAC #942.05) contents according to AOAC International [26]; EE (AOAC #2003.05) was analyzed according to AOAC International [27]. The GE content was determined using an adiabatic calorimetric bomb (C7000; IKA, Staufen, Germany). Chitin was analyzed as D-Glucosamine [28] using a modification of the method described by Madrid et al. [29] for AA.

The AA composition of HI larvae meal and experimental diets is shown in Table 2. AA determination was performed according to the method described in De Marco et al. [5]. After a 22 h hydrolysis step in 6 N HCl at 112 °C under a nitrogen atmosphere, the AA content in hydrolysate was determined by means of HPLC after postcolumn derivatization. Performic acid oxidation occurred prior to acid hydrolysis for methionine and cysteine. Tryptophan was not determined.

**Table 2:** Amino acid (AA) concentration (% of protein) of HI larvae meal and experimental diets

Items	HI larvae meal	HI0	HI25	HI50
Essential AA				
Arginine	3.9	6.3	5.7	5.0
Histidine	2.2	2.4	2.4	2.3
Isoleucine	3.3	4.2	4.0	3.7
Leucine	5.2	7.2	6.7	6.2
Lysine	3.8	7.4	6.5	5.6
Methionine	2.1	2.7	2.4	2.1
Cysteine	0.1	0.9	0.7	0.5
Phenylalanine	3.0	3.9	3.7	3.5
Tyrosine	4.8	3.0	3.5	3.9
Threonine	3.1	4.1	3.8	3.6
Valine	4.9	4.9	4.9	4.9
Non essential AA				
Alanine	6.2	6.1	6.2	6.2
Aspartic acid	6.7	9.0	8.5	7.8
Glycine	4.2	1.0	1.7	2.6
Glutamic acid	8.8	7.2	7.6	8.0
Proline	5.5	12.4	10.6	9.0
Serine	3.7	4.2	4.0	3.9

The FA composition of HI larvae meal and of the experimental diets was assessed using the method described by Schmid et al. [30]. Fatty acid methyl esters (FAME) were separated, identified and quantified on the basis of the chromatographic conditions reported by Renna et al. [31]. The results were expressed as g/100 g of total detected fatty acids (TFA) (Table 3).

**Table 3:** Fatty acid composition (g/100 g of TFA) of HI larvae meal and experimental diets

Items	HI larvae meal	HI0	HI25	HI50
C10:0	1.32	0.00	0.16	0.31
C12:0	54.59	0.12	14.76	28.27
C14:0	10.14	5.78	7.25	8.39
C14:1 *c* + C15:0	0.51	0.57	0.56	0.53
C15 iso	0.01	0.21	0.16	0.11
C16:0	12.03	18.84	17.92	16.52
C16 iso	0.02	0.09	0.08	0.06
C16:1 *c*	3.94	6.23	3.73	3.42
C17 iso	0.02	0.56	0.41	0.28
C17 aiso	0.10	0.29	0.27	0.24
C17:1 *c*9	0.08	0.28	0.22	0.19
C18:0	1.77	4.66	3.96	3.17
C18:1 *t*	0.09	0.18	0.14	0.12
C18:1 *c*9	7.98	22.47	19.46	16.46
C18:1 *c*11	0.28	3.85	3.05	2.02
C18:2 n6	5.98	8.68	8.07	7.34
C18:3 n6	0.05	3.33	2.46	1.58
C18:3 n3	0.79	2.31	1.94	1.55
C20:0	0.10	0.28	0.27	0.18
C20:1 *c*9	0.00	0.15	0.13	0.08
C20:1 *c*11	0.00	0.30	0.21	0.15
C20:2 n6	0.02	1.95	1.34	0.86
C20:3 n6	0.00	0.14	0.10	0.06
C20:4 n6	0.00	0.75	0.53	0.33
C20:5 n3	0.00	7.83	5.59	3.37
C22:0	0.03	0.13	0.12	0.09
C22:1 n9	0.00	2.73	1.96	1.21
C22:5 n3	0.00	1.53	1.10	0.63
C22:6 n3	0.00	5.78	4.07	2.50
Σ SFA	80.28	30.96	45.35	57.61
Σ MUFA	12.88	36.75	29.46	24.17
Σ PUFA	6.84	32.29	25.19	18.22
Σ PUFA/Σ MUFA	0.09	1.04	0.56	0.32
Σ n3	0.79	17.45	12.69	8.05
Σ n6	6.05	14.83	12.50	10.17
Σ n3/Σ n6	0.13	1.18	1.02	0.79

*Abbreviations*: *HI Hermetia illucens*, *c* cis, *t* trans, *SFA* saturated fatty acids, *MUFA* monounsaturated fatty acids, *PUFA* polyunsaturated fatty acids, *TFA* total fatty acids

All values are reported as mean of triplicate analyses

### Digestibility trial

An in vivo digestibility experiment was performed to determine the apparent digestibility coefficients (ADC) of the diets.

Thirty-six trout (weight 182.4 ± 8.3 g) were divided into twelve 40-L cylindroconical tanks connected to the same open water system of the growth trial. After 14 days of acclimatization with the experimental diets, the fish were fed by hand to visual satiety twice a day. The apparent digestibility coefficients were measured using the indirect acid-insoluble ash method; 1% celite® (Fluka, St. Gallen, Switzerland) was added to the diets as inert marker in substitution of 1% of starch gelatinized (D500). The faeces were collected daily from each tank for three consecutive week, using a continuous automatic device, as described by Palmegiano et al. [32]. The faeces were freeze dried and frozen (−20 °C) until analyzed. The ADC of DM (ADC_DM_), crude protein (ADC_CP_), ether extract (ADC_EE_) and gross energy (ADC_GE_) were calculated following Palmegiano et al. [32].

### Growth trial

#### Fish and rearing conditions

A 78-day trial was carried out on three hundred sixty mixed-sex rainbow trout purchased from a private fish hatchery (“Troticoltura Bassignana”; Cuneo, Italy). At the beginning of the trial, fish were lightly anesthetised (MS-222; PHARMAQ Ltd., UK), individually weighed (178.9 ± 9.81 g) and randomly divided into twelve indoor fiberglass tanks of 1 m^3^ (four replicate tanks per diet). Artesian well water (constant temperature of 13 ± 1 °C) was supplied in flow-through open system with each tank having a water inflow of 8 L/min. Dissolved oxygen was measured every fortnight and ranged between 7.6 and 8.7 mg/L. Feed was distributed by hand twice a day, 7 days per week. The first week, the daily quantity of feed distributed was set at 1.2% of the tank biomass and it was then increased at 1.5%. Feed intake was checked at each administration: all the supplied feed was consumed and no feed refusals were recorded during the trial. In order to update the daily FR, the biomass tanks were weighed in bulk every 14 days. Mortality was checked every day.

### Growth performance

At the end of the trial, after 1 day of starving, all fish were individually weighed and the following performance indexes were calculated:

- Survival (%) = 100 – [(number of dead fish/number of fish at start) × 100]
- Weight gain (WG, g) = FBW (final body weight, g) – IBW (initial body weight, g)
- Specific growth rate (SGR, %/d) = [(lnFBW – lnIBW)/number of feeding day] × 100
- Feed conversion ratio (FCR) = total feed supplied (g, DM)/WG (g)
- Protein efficiency ratio (PER) = WG (g)/total protein fed (g, DM)
- Feeding rate (FR, %/d) = [(total feed supplied (g, DM) × 100/number of feeding day)]/[e^(lnFBW + lnIBW) × 0.5^].

### Condition factor, somatic indexes and fillet physical quality parameters

Twenty fish per treatment (five fish per tank) were killed by over anaesthesia. An image of each specimen was recorded with a digital camera (Nikon D3100; Minato, Tokyo, Japan) and fish length was measured using Image-Pro Plus 5.1 software (Media Cybernetics Inc., Bethesda, Rockville, MD, USA) to determine the Fulton’s condition factor (K). The fish were dissected to determine the carcass yield (CY), the hepatosomatic index (HSI), the viscerasomatic index (VSI) and the coefficient of fatness (CF). The somatic indexes were obtained as follows:

- K = [fish weight (g)/(body length)^3^ (cm)] × 100
- CY (%) = [total weight without gut and gonad (g)/fish weight (g)] × 100
- HSI (%) = [liver weight (g)/fish weight (g)] × 100
- VSI (%) = [gut weight (g)/fish weight (g)] × 100
- CF (%) = [perivisceral fat weight (g)/fish weight (g)] × 100

The fish carcasses were then refrigerated (+4 °C) and physical parameters determined 24 h after death. The fish were filleted and the muscle pH (pH_24_) was measured on each right dorsal fillet using a Crison MicropH 2001 (Crison Instruments, Barcelona, Spain) equipped with a combined electrode and an automatic temperature compensator. The flesh colour was assessed on the inside portion of each left dorsal fillet using a bench colorimeter ChromaMeter CR-400 Konica Minolta Sensing (Minolta Sensing Inc., Osaka, Japan). The results were expressed in terms of lightness (L*), redness (a*) and yellowness (b*) in the CIELAB colour space model [33]. Two colour readings were taken on each fillet and averaged.

### Fillet chemical composition and fatty acid profile

The left dorsal fillets of eight fish per treatment (two fish per tank) were finely ground (cutting mill MLI 204; Bühler AG, Uzwil, Switzerland) and freeze dried (Edwards MF 1000, Milan, Italy). DM, CP, EE, ash contents and the FA composition of the fillets were determined as described in Belforti et al. [34]; FAME peaks were identified by injecting pure FAME standards as detailed by Renna et al. [35]. Fillet chemical composition was expressed as g/100 g wet weight (WW), while FA results were expressed as g/100 g of TFA.

The atherogenicity (AI) and thrombogenicity (TI) indexes of fish muscles were calculated. In these indexes, different weights are attributed to various categories of fatty acids in relation to their different contribution to the prevention or promotion of coronary heart diseases and precisely:

$$ \mathrm{AI}=\left(\mathrm{aC}12:0+\mathrm{bC}14:0+\mathrm{cC}16:0\right)/\left(\mathrm{dP}+\mathrm{eM}+{\mathrm{fM}}^{'}\right) $$

where: P is the sum of n3 and n6 PUFA; M is the oleic acid and M´ is the sum of other monounsaturated fatty acids (MUFA); a, b, c, d, e, f are empirical constant; b = 4 and other constants = 1.

$$ \mathrm{TI}=\left(\mathrm{C}14:0+\mathrm{C}16:0+\mathrm{C}18:0\right)/\left[\left(\mathrm{n}\mathrm{M}+{\mathrm{oM}}^{'}+\mathrm{p}\left(\mathrm{n}6\right)+\mathrm{q}\left(\mathrm{n}3\right)+\left(\mathrm{n}3/\mathrm{n}6\right)\right.\right] $$

where M and M´ are as before; n, o, p, q are empirical constants; n, o, p = 0.5 and q = 3.

### Morphometric investigations

At the end of the growth trial, morphometric investigations on intestinal tract were made on sixteen fish per treatment (four fish per tank). Anterior intestinal segment samples (approximately 5 cm in length) were excised and flushed with 0.9% saline to remove all the contents. Gut segments were fixed in 10% buffered formalin solution, routinely embedded in paraffin wax blocks, sectioned at 5 μm thickness, mounted on glass slides and stained with Haematoxylin & Eosin. Morphometric analysis using the Image Pro-Plus software were performed for each fish on 10 well-oriented and intact villi. The evaluated morphometric index was the villus height (from the tip of the villus to the submucosa).

### Statistical analyses

Data were analyzed by one-way ANOVA using IBM SPSS Statistics 20.0. The following model was used:

$$ {\mathrm{Y}}_{\mathrm{i}\mathrm{j}}=\upmu +{\mathrm{D}}_{\mathrm{i}}+{\upvarepsilon}_{\mathrm{i}\mathrm{j}} $$

where Y_ij_ = observation; μ = overall mean; D_i_ = effect of diet (HI0, HI25, HI50); ε_ij_ = residual error.

The Kolmogorov–Smirnov test was used to check dependent variables for normality. The assumption of equal variances was assessed by Levene’s homogeneity of variance test. If such an assumption did not hold, the Brown-Forsythe statistic was performed to test for the equality of group means instead of the F one. Pairwise multiple comparisons were performed to test the difference between each pair of means (Tukey’s test and Tamhane’s T2 in the cases of equal variances assumed or not assumed, respectively).

The results were expressed as mean and pooled standard error of the mean (SEM). Significance was declared at *P* ≤ 0.05

## Results

### Diets

All diets were comparable in terms of DM and other main nutrients. The GE ranged between 21.71 and 22.60 MJ/kg DM. The AA compositions of HI larvae meal and the three assay diets are presented in Table 2. In the HI meal among essential AA (EAA), leucine, tyrosine and valine were the most abundant, whereas glutamic and aspartic acid were the most abundant non essential AA. HI larvae meal reported similar values for histidine and lower values for arginine and lysine than FM [36]. Concerning diet EAA profile, tyrosine increased at the increasing dietary HI inclusion levels. Conversely, all the other EAA decreased except histidine and valine that remain constant. As far as the FA composition is concerned (Table 3), lauric acid (C12:0) was by far the most represented FA in HI larvae meal (54.59 g/100 g of TFA). Consequently, in the experimental diets lauric acid and total saturated fatty acids (SFA) increased following the increased inclusion of insect meal. Arachidonic (C20:4 n6), eicosatrienoic (C20:3 n6), eicosapentaenoic (EPA, C20:5 n3), docosapentaenoic (DPA, C22:5 n3) and docosahexaenoic (DHA, C22:6 n3) acids were not detected in the HI larvae meal; their contents consequently decreased in the experimental diets with the increase of insect meal inclusion level. Such decreases have also to be imputed to the contemporary decrease in the fish oil content of the diets. The total n3 and n6 contents in the diets decreased from 17.45 (HI0) to 8.05 (HI50) and from 14.83 (HI0) to 10.17 (HI50) g/100 g of TFA, respectively. The ∑ n3/∑ n6 FA ratio decreased in the diets following the increase of HI larvae meal inclusion.

### Digestibility trial

The ADC values of nutrients are presented in Table 4. Differences (*P* < 0.05) were recorded between HI25 and HI50 for DM and CP digestibility. In both cases, HI25 showed the highest values.

**Table 4:** Apparent digestibility coefficient of dry matter, proteins, ether extract and gross energy of rainbow trout fed the experimental diets (*n* = 4)

Items	HI0	HI25	HI50	SEM	*P*-value
ADC_DM_	0.76 ab	0.79 a	0.74 b	0.009	0.012
ADC_CP_	0.89 ab	0.91 a	0.87 b	0.006	0.023
ADC_EE_	0.97	0.99	0.97	0.005	0.245
ADC_GE_	0.60	0.65	0.60	0.012	0.100

*Abbreviations*: *HI Hermetia illucens*, *SEM* standard error of the mean, *P* probability, *ADC*_*DM*_ dry matter apparent digestibility coefficient, *ADC*_*CP*_ crude protein apparent digestibility coefficient, *ADC*_*EE*_ ether extract apparent digestibility coefficient, *ADC*_*GE*_ gross energy apparent digestibility coefficient

Different letters within a row indicate significant differences (*P* ≤ 0.05)

### Growth trial

#### Growth performance

The survival (%) and growth performance parameters are reported in Table 5. No differences (*P* > 0.05) were observed for the considered parameters. Fish survival ranged from 97.4% (HI0) to 100% (HI50). The fish readily accepted the experimental diets. All feeds were consumed without rejection or loss. IBW was comparable among the treatments and in 78 days the fish tripled their BW. FCR remained below 1 in all treatments.

**Table 5:** Survival and growth performances of rainbow trout fed the experimental diets (*n* = 4)

Items	HI0	HI25	HI50	SEM	*P*-value
Survival,%	97.4	98.3	100.0	0.681	0.298
IBW,g	178.9	178.8	179.1	0.099	0.579
FBW,g	539.3	545.4	538.0	5.098	0.849
WG,g	360.5	366.5	358.9	5.079	0.840
SGR,%/d	1.40	1.42	1.41	0.013	0.935
FCR	0.90	0.88	0.90	0.009	0.739
PER	2.46	2.52	2.47	0.024	0.579
FR,%/d	1.33	1.32	1.33	0.005	0.442

*Abbreviations*: *HI Hermetia illucens*, *SEM* standard error of the mean, *P* probability, *IBW* initial body weight, *FBW* final body weight, *WG* weight gain, *SGR* specific growth rate, *FCR* feed conversion ratio, *PER* protein efficiency ratio, *FR* feeding rate

### Condition factor, somatic indexes and fillet physical quality parameters

No differences were highlighted for condition factor, somatic indexes, carcass characteristics and physical quality traits (pH and color) of the fillets (Table 6).

**Table 6:** Condition factor, somatic indexes and fillet physical quality parameters of rainbow trout fed the experimental diets (*n* = 20)

Items	HI0	HI25	HI50	SEM	*P*-value
K	1.18	1.23	1.21	0.011	0.198
CY	88.88	88.87	88.94	0.110	0.968
HSI	1.63	1.73	1.71	0.021	0.158
VSI	8.94	9.25	9.21	0.141	0.632
CF	1.57	1.41	1.39	0.066	0.472
Quality parameters					
pH_24_	6.36	6.38	6.41	0.016	0.557
L*	45.06	46.38	47.98	0.618	0.157
a*	0.24	−0.25	−0.29	0.187	0.434
b*	6.34	6.68	6.67	0.145	0.575
Hue	−0.48	−0.09	−0.41	0.179	0.644
Chroma	6.50	6.86	6.77	0.153	0.608

*Abbreviations*: *HI Hermetia illucens*, *SEM* standard error of the mean, *P* probability, *K* condition factor, *CY* carcass yield, *HSI* hepatosomatic index, *VSI* viscerosomatic index, *CF* coefficient of fatness, *pH*_*24*_ fillet muscle pH at 24 h post mortem, L* lightness, a* redness, b* yellowness

### Fillet chemical composition and fatty acid profile

The fillet chemical and FA compositions of the fish fed the experimental diets are reported in Table 7. Regarding the chemical composition, only CP remained unaffected by treatment. The inclusion of HI larvae meal progressively increased DM and EE contents in HI25 and HI50 compared to HI0 (*P* < 0.05). The ash content was higher in HI0 (1.20 g/100 g WW) compared to HI25 (1.00 g/100 g WW), while HI50 showed intermediate values.

**Table 7:** Fillets chemical and fatty acid compositions of rainbow trout fed the experimental diets (*n* = 8)

Items	HI0	HI25	HI50	SEM	*P*-value
Chemical composition, g/100 g WW					
DM	25.06 b	25.79 ab	26.31 a	0.194	0.022
CP	19.58	19.37	19.56	0.064	0.360
EE	4.18 b	5.19 ab	5.48 a	0.219	0.036
Ash	1.20 a	1.00 b	1.09 ab	0.030	0.015
Fatty acid composition, g/100 g of TFA					
C12:0	0.29 c	8.07 b	14.66 a	1.239	0.000
C14:0	3.97 c	5.49 b	6.47 a	0.224	0.000
C14:1 *c* + C15:0	0.40	0.47	0.43	0.014	0.072
C16:0	22.73 a	20.69 b	20.38 b	0.280	0.000
C16:1 *c*	6.22	6.45	6.41	0.075	0.428
C17:0	0.49 a	0.41 b	0.35 b	0.015	0.000
C17:1 *c*9	0.05 b	0.08 a	0.07 ab	0.004	0.002
C18:0	5.45 a	4.79 b	4.64 b	0.107	0.001
C18:1 *t*	0.97 a	0.69 b	0.66 b	0.044	0.001
C18:1 *c*9	31.33 a	27.62 b	26.37 b	0.531	0.000
C18:1 *c*11	4.03 a	3.43 b	2.91 c	0.102	0.000
C18:2 n6	10.30 a	9.46 ab	9.01 b	0.208	0.027
C18:3 n6	3.05 a	2.29 b	1.73 c	0.135	0.000
C18:3 n3	1.97 a	1.90 a	1.49 b	0.066	0.002
C20:0	0.36 a	0.36 a	0.23 b	0.016	0.000
C20:1 *c*11	0.40 a	0.39 a	0.25 b	0.018	0.000
C20:2 n6	0.83 a	0.74 a	0.57 b	0.030	0.000
C20:3 n6	0.30	0.27	0.29	0.007	0.442
C20:4 n6	0.49 a	0.46 a	0.29 b	0.022	0.000
C20:5 n3	2.33 a	2.03 a	1.07 b	0.133	0.000
C22:5 n3	0.66 a	0.50 b	0.23 c	0.038	0.000
C22:6 n3	3.39 a	3.40 a	1.50 b	0.203	0.000
∑ SFA	33.28 c	39.81 b	46.74 a	1.186	0.000
∑ MUFA	43.40 a	39.13 b	37.09 c	0.623	0.000
∑ PUFA	23.31 a	21.06 b	16.17 c	0.693	0.000
∑ PUFA/∑ SFA	0.70 a	0.53 b	0.35 c	0.033	0.000
∑ n3	8.34 a	7.84 a	4.29 b	0.418	0.000
∑ n6	14.97 a	13.22 b	11.88 c	0.309	0.000
∑ n3/∑ n6	0.56 a	0.59 a	0.36 b	0.024	0.000
AI	0.58 c	0.84 b	1.15 a	0.050	0.000
TI	0.60 b	0.62 b	0.85 a	0.026	0.000

*Abbreviations*: *HI Hermetia illucens*, *SEM* standard error of the mean, *WW* wet weight, *DM* dry matter, *CP* crude protein, *EE* ether extract, *c* cis, *t* trans, *FA* fatty acids, *SFA* saturated fatty acids, *MUFA* monounsaturated fatty acids, *PUFA* polyunsaturated fatty acids, *AI* atherogenicity index, *TI* thrombogenicity index, *TFA* total fatty acids

Different letters within a row indicate significant differences (*P* ≤ 0.05)

All values are reported as mean of triplicate analyses

Concerning the fillet FA composition, C12:0 sharply increased (from 0.29 to 8.07 and 14.66 g/100 g of TFA in HI0, HI25 and HI50 groups, respectively), showing highly significant differences among all treatments. Increasing trends were also observed for myristic acid (C14:0) and the total SFA content, which were both higher in HI25 compared to HI0 and in HI50 compared to HI25. The majority of individual MUFA were also affected by diet. Most of them showed a decreasing trend following the increase of HI larvae meal inclusion. Regarding PUFA, the total content decreased and showed highly significant differences among all treatments. Individual PUFA showed a decreasing trend from HI0 to HI50, with the exception of eicosatrienoic acid (C20:3 n6) which was not affected by diet. EPA and DHA showed lower contents in HI50 compared to HI0 and HI25, while DPA was already reduced at the lower inclusion level of HI meal. Overall, lipids health indexes such as the Σ PUFA/Σ SFA ratio, the Σ n3/Σ n6 FA ratio, AI and TI were negatively affected by HI larvae meal inclusion in the diet. The Σ PUFA/Σ SFA ratio and AI already worsened with the lower dietary HI larvae meal inclusion level, while for the Σ n3/Σ n6 FA ratio and TI significant variations compared to the control diet were only observed with the higher dietary level of insect meal.

### Morphometric investigations

Dietary HI inclusion did not affect the intestinal morphology of the trout of the present study (HI0 = 1.48 mm; HI25 = 1.49 mm; HI50 = 1.60 mm; SEM = 0.039; *P* > 0.05).

## Discussion

### Digestibility trial

As far as digestibility is concerned, differences were only found for ADC_DM_ and ADC_CP_ between HI25 and HI50, while HI0 showed intermediate values. The lowest values were recorded for HI50 (0.74 and 0.87 for DM and CP, respectively). Nevertheless, it has to be highlighted that in all treatments the digestibility of these nutrients was very good and higher than values reported for trout fed bacterial protein meal [37]. The good ADC values found in our trial support the growth performance results.

The lower levels of DM and CP digestibility in HI50 compared to HI25 could be attributed to the higher level of chitin supplied by the HI50 meal. These findings could be ascribed to the proteins linked to chitin and therefore present in the insect exoskeleton [38]. Even if gastrointestinal bacteria and chitinase activity were reported in some fish species [11], the chitin digestibility is usually very low or completely absent in rainbow trout [6]. Marono et al. [39] indicated that chitin is the main factor affecting the in vitro protein digestibility of HI meal and showed that CP digestibility was negatively correlated to the chitin content of the meal. Moreover, it has been shown that high concentrations (up to 45%) of the chitin present in the cuticular exoskeleton of insects negatively affected feed intake and reduced protein digestibility [40]. Despite the decrease of CP digestibility in HI50 compared to HI25, no negative effects on trout growth performance were highlighted in this trial. Marono et al. [39] estimated that chitin content of different HI samples varied from 2.86 to 5.50% of the meal (average: 4.25%). In our trial, a dietary inclusion of 20 or 40% of HI, replacing 25 or 50% of dietary FM, would thus have brought a maximal amount of 2% of chitin likely not being able to produce negative effects on trout performances.

No differences were found in our trial regarding ADC_EE_ and ADC_GE_ among treatments. Lock et al. [12] reported that the dietary inclusion of insect meal did not affect the FA digestibility in Atlantic salmon. These authors reported that the FA digestibility ranged between 82.5 and 100% and underlined how lauric acid remained highly digestible even in diets where it was highly abundant.

In comparison to other alternative animal protein sources, the ADC_CP_ values found in our trial were higher than those observed for feather meal or meat and bone meal (81 and 83%, respectively) for rainbow trout [4].

### Growth trial

#### Growth performance

The results of the growth trial showed that the inclusion of 20 and 40% of a partially defatted HI larvae meal in substitution of 25 and 50% of FM in trout diets did not lead to any adverse effect on growth performance. The trout (all treatments merged) showed an average WG of about 4.7 g/d, indicating a good nutrient utilization. Such result is also supported by the very favourable FCR (all less than 1), the high PER (always greater than 2.4), and a SGR near to 1.4. No palatability problems were highlighted and the dietary inclusion of 20 and 40% of HI reduced the diet FM component from 60 to 45 and 30% respectively.

The results obtained in this trial do not always agree with those reported in other studies performed using HI. Indeed, St-Hilaire et al. [8], using a full fat HI prepupae meal, observed a decrease in WG and a worsening in FCR when HI prepupae meal was included in trout diets for more than 15% (FM substitution of more than 25%). It has to be highlighted that the initial body weight of trout used in the trial performed by St-Hilaire et al. [8] was 22.2 g and that young fish have higher requirements than adult fish as those used in the present trial. The huge difference in fish weight may partially explain the differences observed in growth performances between trials. St-Hilaire et al. [8] ascribed the reduced performances to a lower energy intake in fish fed insects compared to fish fed control diet. In our trial, the experimental diets contained equal amounts of GE. Moreover, the replacement of 50% of the FM component in St-Hilaire et al. [8] trial reduced to only 18% the FM dietary inclusion and, even if not suggested by authors, this could have led to a deficit in EAA.

Sealey et al. [9] used full-fat HI prepupae meals obtained from larvae reared on manure or manure enriched with fish by-products substrates. The FM substitution was 25 and 50% for both HI meals, leading to dietary inclusion rates of about 16 and 32% for the former and of about 18 and 36% for the latter HI meal. Feeding rainbow trout of similar weight of those of the current trial (about 145 g), these authors showed that WG of trout fed the enriched diets was lower but not different from that of trout fed the control diet, while it was reduced in trout fed the non-enriched HI diets. In both cases, no significant differences were reported for feed consumption and FCR. In addition to the use of a defatted HI meal, the differences observed in our study compared to the one of Sealey et al. [9], could be related to the HI meal nutrient availability. Indeed, Sealey et al. [9] suggested a lower nutrient availability in HI diets supporting their statement with a lower muscle lipid in fish fed both normal and enriched HI meals compared to fish fed the FM diet. In our trial an increase in lipid muscle content was reported suggesting a good nutrient availability.

In a study on rainbow trout fed diets containing 0, 50 and 75% of full-fat HI meal in substitution of FM, Stamer et al. [41] concluded that the substitution up to 50% may be possible without negative effects on WG, FCR and PER. However, it has to be noted that, according to the information reported in Stamer et al. [41], it is difficult to understand the levels of inclusions of HI meal in the different treatments since no quantitative data regarding formulations is produced and it is not possible to extrapolate the quantity of FM used in control diet.

Kroeckel et al. [19], in a trial with turbot juveniles, formulated six diets containing increasing levels of HI meal (CP: 54.1% DM; EE: 13.4% DM). These authors found a decrease of feed intake while increasing HI incorporation due to low diet palatability. They also noted that growth performance was affected by dietary HI inclusion and reported lower FBW and SGR in all treatments containing HI, whereas FCR was worsened only at HI inclusion levels higher than 33%. Similarly to Sealey et al. [9], Kroeckel et al. [19] reported a decrease in final body crude lipid content in fish fed HI diets (significant only over 33% of inclusion) denoting a decrease in nutrient availability as also supported by the low digestibility coefficients found for HI meal. These authors attributed the low lipid utilization and the small lipid retention at higher HI inclusion levels to the presence of chitin and its negative influence on lipid digestibility. Chitin contents in HI25 and HI50 diets used in the current trial (1 and 2% respectively) resulted lower than those reported by Kroeckel et al. [19] and these contents seem not sufficient to induce negative effects on trout performances.

Finally, Lock et al. [12] carried out a trial with salmon where FM (200 g/kg diet) was substituted by 25, 50 and 100% HI (inclusion of 5, 10 and 20% HI in the experimental diets). Their results showed that feed intake decreased moderately while increasing HI inclusion; however, FCR decreased resulting in an equal net growth of the fish.

Contrasting results of the over cited studies could also be ascribed to the type of insect meal, the level of fat in meals, the fish species and the fish age [6].

### Condition factor, somatic indexes and fillet physical quality parameters

The Fulton’s condition factor is used to compare the condition, fatness, or wellbeing of fish, based on the assumption that heavier fish of a given length are in better condition [42], K values less than 1 imply that fish are not in good state of well-being within their habitat, while values greater than 1 imply that fish are in good physiological state of well-being. The K values reported in our trial, independently from the treatment, were greater than 1 and were similar to those recorded in large rainbow trout fed a plant protein mixture [43]. These authors explained also that in trout a higher condition factor is mainly due to a high mesenteric fat content, suggesting higher fat synthesis and deposition. In our study, trout fed HI meal diets showed no differences both for K and CF, therefore is reasonable that insect meal did not modify fat synthesis and deposition of the fish fed experimental diets.

As far as HSI is concerned, the obtained values were higher than those reported by Sealey et al. [9], which ranged between 1.1 to 1.2, and no differences were found as a consequence of dietary inclusion of HI. In trout fed by graded levels of canola oil, Dernekbaşi [44] ascribed altered values of HSI to metabolic problems or liver deficiencies. The lack of HSI differences found in our trial among treatments seems to confirm the well-being status of the fish and that no problems arose by the ingestion of HI. In our trial, VSI did not differ among treatments. Such results partly differ from what reported by Lock et al. [12] who found different HSI and VSI values in Atlantic salmon fed diets containing two different HI meals if compared to a FM-based diet; the results obtained by Lock et al. [12] were however dependent on type of insect meal used and level of inclusion in the diet. Using *Tenebrio* molitor larvae meal in rainbow trout, Belforti et al. [34] found significant differences for HSI values, with a decrease of this index at the increase of insect meal levels in the diets, while no significant differences were observed for VSI among groups.

A decrease in nutrient availability is often associated to a decrease in the intraperitoneal fat ratio [9]. Similarly, some authors imputed to a low nutrient digestibility the decrease in CY when fish were fed FM substitutes [45, 37].

Variations in fillet pH values could be due to several factors like culture density, stress level prior to killing, dietary treatments and fillet energy storage [45]. Moreover, high pH could negatively influence the shelf life of the product because an alkaline environment is not able to inhibit microbial degradation processes. The magnitude of the pH decrease is species specific and in this study the values obtained for pH at 24 h post mortem are in line with those reported for trout fed two levels of lipids [46] and lower than those found by Gai et al. [37] in trout fed bacterial or plant protein meals. Fillet colour is a very important quality parameter since it is directly perceived by the consumer. In our trial, the use of HI did not influence colour parameters. To the best of our knowledge, no published data are currently available comparing fillet colour of trout fed HI meals and other diets. Trout fed plant proteins sometimes highlighted increased b* that could partially be attributed to the presence in the feeds of plant pigments like corn carotenoids [43]; this could negatively influence the consumer acceptance of the fillets [37, 45]. A direct relationship between fillets brightness and their lipid content was demonstrated in salmonids [47]. However, in our trial, no differences were recorded for L* (*P* = 0.157), despite the observed EE increase in fillet following the dietary inclusion level of insect meal. Finally, a significant decrease for L* was reported by Pieterse et al. [48] in breast muscle of broilers fed diets containing *Musca domestica* larvae meals compared to a FM based diet.

### Fillet chemical composition and fatty acid profile

The use of HI did not affect the net content of proteins in trout fillets. The obtained CP values are consistent with those found by Palmegiano et al. [32] and Gai et al. [37], who used alternative protein sources (rice protein concentrate meal and bacterial protein meal or pea protein concentrate, respectively) in rainbow trout feeds. Similarly, no change in the fillet protein content with the use of HI was obtained by Sealey et al. [9].

In the present study, the observed EE increase in fillet indicates a good availability and storage of nutrients in the trout fed HI. Such finding disagree with other authors [9, 19, 34] who reported decreased values of DM and EE in the fillets of fish fed insect meals, probably due to a decrease in nutrient availability.

So far, this is the first trial on rainbow trout dealing with partially defatted HI. Former trials evaluated HI prepupae meals having an average fat content of about 30% [8, 9]. The FA profile of the partially defatted HI larvae meal used in this trial showed differences if compared to the ones used in researches performed by St-Hilaire et al. [8] or by Sealey et al. [9]. Differences could be due to the rearing substrates used. Indeed, while the present HI was obtained from larvae reared on vegetable by-products, the ones of St-Hilaire et al. [8] and of Sealey et al. [9] were obtained rearing black soldier fly on swine manure. The FA profile of fish usually mirrors that of the administered diets; as a consequence, a noticeable influence of HI on the fillet FA composition was observed in our trial. The content of lauric acid differed among all treatments, with values 27.8 and 50.6 times higher in the trout fed HI25 and HI50 compared to HI0, respectively. Even if to a lesser extent, this increase was also noticed for C14:0. The rise in lauric and myristic acids led to increased total SFA in the fillets of the trout fed HI diets if compared to those fed the control diet. As recently observed by Li et al. [17] in juvenile Jian carp (*Cyprinus carpio* var. Jian) fed HI larvae oil, we also found relatively lower levels of lauric acid in the muscles of HI-fed trout if compared to the respective lauric acid dietary levels, which may suggest readily utilization of this FA for energy production.

Conversely, for valuable very long chain n3 PUFA (such as EPA and DHA), well known for their beneficial effects on human health, differences were only found between HI50 and the other two treatments. Such result could be due to the still high fish oil content of the HI25 diet and/or could suggest good elongation and desaturation activities in the HI25 group, probably due to a stimulation of the involved lipogenic enzymes, as suggested by Figueiredo-Silva et al. [49] and also reported by Lock et al. [12]. These results only partially agree with the findings of Belforti et al. [34] who found significant reductions of EPA and DHA in trout fillets starting from the lowest level of FM substitution with full-fat *T. molitor* meal. Similarly, the Σ n3/Σ n6 FA ratio did not differ between HI0 and HI25, modulating the negative effects on fish nutritional values when alternative proteins are used in rainbow trout feeds [9, 32, 34].

Fish is considered as a valuable food to prevent coronary heart diseases due to its high levels of PUFA. Indexes (AI, TI) correlating the different amount of some specific SFA, MUFA and PUFA of both the n3 and n6 series were proposed to indicate the contribution of these FAs to the prevention or promotion of pathological phenomena. Series n3 and n6 MUFA and PUFA are considered to have similar activity towards the prevention of thrombi onset, while n3 PUFA seem to be more important in the prevention of atheroma insurgence. There were differences in AI and TI of the fillets from the trout fed diets with different HI replacement levels. Anyway, the obtained values were less or around 1.0, still considered to be healthy for human consumers.

### Morphometric investigations

Dietary HI inclusion did not induce morphological changes in the rainbow trout intestine, thus suggesting no negative influence on the intestinal physiological development. Our results are similar to what observed by Lock et al. [12], who found normal histology of the midgut epithelium in salmon fed insect larvae meals. Similar data were also obtained by Biasato et al. [50] who recently evaluated the histomorphological alterations in free-range chickens fed diets with *T. molitor* larvae meal inclusion, finding no differences related to insect meal utilization.

Despite lacking of information about gut morphometry in fish, in other species long villi have been reported to be associated with an increased population of beneficial bacteria in the gut [51]. Indeed, lengthening of villi may increase villus absorptive area, with subsequent satisfactory digestive enzyme action and greater transport of nutrients [52].

## Conclusions

This study provided new data and knowledge on the potential use of a new sustainable feedstuff for carnivorous fish diets. The ADC values found in this trial were overall satisfactory. Main findings indicated that dietary FM can be replaced by a partially defatted HI larvae meal up to 50% of substitution (40% of inclusion in diet) without negative effects on growth performance, condition factor, somatic indexes, physical quality parameters and gut morphology. The fillet chemical composition was influenced by the use of insect meal with the exception of CP content. From the lipid point of view, HI exerted negative effects both on EE content and FA composition. Nevertheless, it can be stated that up to 20% of HI dietary inclusion, no negative effects were reported as far as the contents of beneficial very long chain n3 FA (EPA and DHA) are concerned.

Insect meals have recently been authorized for aquafeed. Notwithstanding, commercial business application needs future research. Particularly, information on the influence of insect meals on feed physical characteristics in extruded aquafeeds is still missing. Moreover, insect meal producers still face with the problem of insect meal chitin content. Therefore the assessment of methods to improve the digestibility of insect meals for their correct introduction as raw material in fish feeds are needed.

## Abbreviations

AA: Amino acids
ADC: Apparent digestibility coefficients
AI: Atherogenicity index
BW: Body weight
*c*: *cis*
CF: Coefficient of fatness
CP: Crude protein
CY: Carcass yield
DHA: Docosahexaenoic acid
DM: Dry matter
DMI: Dry matter intake
DPA: Docosapentaenoic acid
EAA: Essential amino acids
EE: Ether extract
EPA: Eicosapentaenoic acid
FA: Fatty acids
FAME: Fatty acid methyl esters
FBW: Final body weight
FCR: Feed conversion ratio
FM: Fish meal
FR: Feeding rate
GE: Gross energy
HI: *Hermetia illucens*
HSI: Hepatosomatic index
IBW: Initial body weight
K: Fulton’s condition factor
MUFA: Monounsaturated fatty acids
PAPs: Processed animal proteins
PER: Protein efficiency ratio
PUFA: Polyunsaturated fatty acids
SBM: Soybean meal
SEM: Standard error of the mean
SFA: Saturated fatty acids
SGR: Specific growth rate
*t*: *trans*
TFA: Total fatty acids
TI: Thrombogenicity index
VSI: Viscerosomatic index
WG: Weight gain
WW: wet weight
